# Efficient cell penetration and delivery of peptide nucleic acids by an argininocalix[4]arene

**DOI:** 10.1038/s41598-019-39211-4

**Published:** 2019-02-28

**Authors:** Jessica Gasparello, Alex Manicardi, Alessandro Casnati, Roberto Corradini, Roberto Gambari, Alessia Finotti, Francesco Sansone

**Affiliations:** 1 Department of Life Sciences and Biotechnology, Section of Biochemistry and Molecular Biology, University of Ferrara, Ferrara, Italy; 2 Department of Chemistry, Life Sciences and Environmental Sustainability, University of Parma, Parma, Italy; 3 Present Address: Department of Organic and Macromolecular Chemistry, Ghent University, Ghent, Belgium

## Abstract

The application of Peptide Nucleic Acids (PNAs), mimics of DNA lacking the sugar-phosphate backbone, for antisense/anti-gene therapy and gene editing is limited by their low uptake by cells. Currently, no simple and efficient delivery systems and methods are available to solve this open issue. One of the most promising approach is the modification of the PNA structure through the covalent linkage of poliarginine tails, but this means that every PNA intended to be internalized must be modified. Herein we report the results relative to the delivery ability of a macrocyclic multivalent tetraargininocalix[4]arene (**1**) used as non-covalent vector for anti-miR-221-3p PNAs. High delivery efficiency, low cytotoxicity, maintenance of the PNA biological activity and ease preparation of the transfection formulation, simply attained by mixing PNA and calixarene, candidate this vector as universal delivery system for this class of nucleic acid analogues.

## Introduction

Peptide nucleic acids (PNAs) are DNA analogues in which the sugar-phosphate backbone is replaced by *N*-(2-aminoethyl)glycine units^1–6^. These molecules efficiently hybridize with complementary DNA and RNA, forming both double helices through Watson-Crick base pairing^6^ and triplexes through Watson-Crick and Hoogsteen hydrogen bonds, the latter being able to perform strand invasion^7,8^. PNAs are also very useful for in cells and *in vivo* DNA and RNA recognition, since they are resistant to DNases and proteases^9,10^, and show higher specificity for the target DNA and RNA sequences with respect to nucleic acids^1–6^. For these reasons, they have been proposed for antisense^6,11,12^ and anti-gene^13–15^ therapy in a number of studies and lately for precise gene editing^16–18^. Recently, the use of PNAs as molecules targeting microRNAs (miR/miRNA) has been firmly demonstrated^19–26^.

One of the critical challenges in PNA technology is their delivery to cells^27^, in particular their low uptake by eukaryotic cells^28–32^. In order to solve this drawback, several approaches have been considered. One of these is conjugation with carrier peptides^33–36^, in particular those sensitive to microenvironment changes^24^; antimiR activity was indeed observed for instance by conjugation of PNAs to polyarginine (poly-R) tails^21,25^, or by modification of the PNA backbone with cationic amino acid side chains^37,38^.

As alternative to the chemical modification, for PNA delivery particularly convenient is the use of carriers able to interact with the cargo in a non-covalent and reversible way. This strategy would allow in principle to make available a system effective with all native PNA sequences that are intended to be transported into cells. In this context, it was actually already explored the delivery of PNAs and their derivatives or analogs with liposomes^39^, polymer nanoparticles^40^ and pseudovirions^41^, and by co-transfection with partially complementary DNA^42^. Inorganic nanocarriers, such as nanozeolites^29^ or mesoporous silica nanoparticles (MSNPs)^28^ have been also used for cellular delivery of PNAs; for MSNPs–mediated PNA delivery an anti-miR activity was demonstrated^28^. However, the preparation of all these systems and the PNA incorporation generally require special and often time consuming procedures.

**Figure 1:**
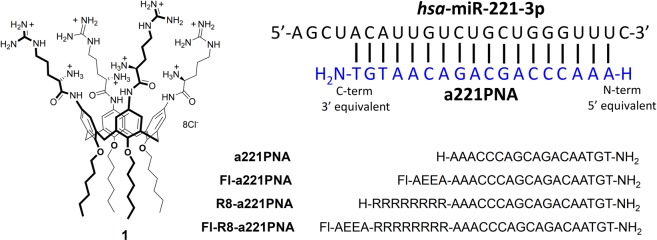
Structure of calix[4]arene **1** and sequences of the PNAs used in the present study. The PNA sequences are complementary to a tract of miR-221-3p as shown up on the right. Modifications at the N-terminus of **a221PNA** were performed linking the report unit and/or the octaarginine sequence. AEEA: 2-(2-aminoethoxy)ethoxyacetyl group, employed as spacer; Fl: Fluorescein.

Calixarenes functionalized with ammonium groups were shown to be suitable for the efficient delivery of nucleic acids. Some of us recently reported^43–46^ on the delivery of plasmid DNA using the macrocyclic calix[4]arene in cone geometry as scaffold for vector molecules. This geometry makes possible the display of two spatially well-defined regions, one hydrophobic and the other hydrophylic, overall resulting in a pronounced amphiphilic character of the vector. The clustering on the macrocycle of only four guanidinium groups or basic amino acids, such as arginine and lysine, was enough to give rise to new non-viral vectors for cell transfection of DNA, more potent than commercial transfecting agents. The exploited parallel arrangement of the amino acid units makes available, with respect to more classical polyarginine peptides, the primary α-amino groups that might favor the protection of the vector–DNA complex from the lysosomal degradation and facilitate the release of DNA from the endosomes into the cytosol through a proton sponge effect. This would, in part, contribute to the high transfection efficiency observed in particular for argininocalix[4]arene **1**^46^ (Fig. 1).

The expected interactions between these cationic calixarenes and DNA can be not only charge-charge interactions between ammonium and phosphates groups, but also hydrogen bonds with nucleobases as previously proposed and shown for analogous positively charged calixarene derivatives^47,48^. Therefore, despite the neutral backbone of PNAs, their similarity with DNA as a consequence of the presence of nucleobases made us reasonably confident that these macrocyclic delivery systems could be also used for the transfection of these mimics. In this work, we herein report that neutral PNAs can be indeed efficiently delivered inside cells using the argininocalix[4]arene **1**, chosen as first example of cationic calixarene to be investigated because of its highest ability to vehicle DNA among a panel of calixarene-based vectors^43–46^. As cellular model system, the human glioma U251 cells were employed. The PNAs used (Fig. 1) were previously demonstrated to be able to target miR-221-3p, thereby altering its biological activity and providing a clear-cut end-point for evaluation of their biological effect using the delivery system under investigation.

## Methods

### Synthesis and characterization of argininocalix[4]arene 1

The structure of the calixarene-based molecule **1** is depicted in Fig. 1. Its synthesis and characterization have been previously reported^46^. Derivative **1**, employed for PNA delivery experiments, was re-suspended in a solution of EtOH/H_2_O/DMSO (2/2/1 v/v/v) under sterile condition.

For a fast evaluation of the interaction with PNA, fluorescently labelled **Fl-a221PNA** (Fl: Fluorescein) was diluted to a final concentration of 2 μM in buffer (HEPES 16 mM, NaCl 40 mM, pH 7.4) and placed in a fluorescence cuvette; to this, a concentrated solution of the argininocalix[4]arene **1** (100 μM in EtOH/H_2_O/DMSO = 2/2/1 v/v/v) was added stepwise (up to a final concentration of 9.9 μM of calixarene), and changes in the fluorescein emission intensity (with λ_ex_ = 494 nm) were measured after each addition. Correction for lamp fluctuation was performed by measuring the fluorescence of a standard 2 μM fluorescein solution after each spectra; this correction and that for dilution were performed using the formula: F_i, corr_ = F_i,obs_ × (F_in_ /F_i_) × (V_i_ /V_in_), where F_i_: intensity at the i_th_ point and F_in_: initial intensity of the fluorescein standard, V_i_: total volume at the i_th_ point and V_in_: initial volume.

### Synthesis and Characterization of a221PNAs

The synthesis of anti-miR-221 PNAs (a221PNAs) was performed using standard manual Boc procedure as previously reported^49^. Purification was performed by HPLC and the PNAs were characterized by HPLC-MS as described previously^49^. PNA features and sequences are reported in Fig. 1.

### Culture Conditions and transfection procedure

The human glioma U251 cells^50^ were cultured in humidified atmosphere of 5% CO_2_/air in RPMI-1640 medium (Gibco, Life Technologies, Monza, Italy) supplemented with 10% (v/v) fetal bovine serum (FBS, Biowest, Nuaillé, France), 100 units/mL penicillin and 100 µg/mL streptomycin (Pen-Strep, Sigma-Aldrich, Saint Louis, Missouri, USA). All PNA transfection procedures were performed under the conditions described here below. Briefly, a mixture constituted by RPMI-1640 medium containing calixarene **1** at 2.5 µM final concentration, and 2 µM PNA was prepared and incubated for 20 minutes at room temperature, without serum. After the incubation, 10% (v/v) of FBS was added. Cell culture medium was removed and replaced with the transfection mixture, which was maintained in contact with cells until the end of the treatment.

### Cell viability assay and effects of calixarene 1 on U251 cell growth

Cell viability after the transfection with **1** was evaluated with Muse Count & Viability Kit (Millipore, Billerica, Massachusetts, USA), using Muse Cell Analyser (Millipore, Billerica, Massachusetts, USA). To perform analysis, 50 µL of suspension cells were added to 225 µL of Muse Count & Viability Reagent, the solution was incubated at room temperature for 5 minutes, protected from the light and then 1 × 10^3^ events were analysed using Muse Cell Analyser (Millipore, Billerica, Massachusetts, USA). The effects on cell growth were studied by determining the cell number/ml using a Z2 Coulter Counter (Coulter Electronics, Hialeah, FL, USA). The effects of calixarene **1** on cell viability, cell growth and cell morphology were compared to those of Lipofectamine RNAiMAX (Invitrogen, Monza, Italy). The concentrations used of lipofectamine were within the range of those suggested by the manufacturer (0.5–12 μl/ml of cell culture).

### FACS analysis

Uptake of fluorescent PNAs was evaluated using FACScan (BD, Becton Dickinson, Franklin Lakes, New Jersey, USA). Cells were washed twice with DPBS 1X, re-suspended in 150 µL of DPBS 1X and analyzed by FACS analysis for FITC fluorescence. For each sample 30.000 events were acquired and data analysis was performed using CellQuest Pro software (BD, Becton Dickinson, Franklin Lakes, New Jersey, USA).

### Cell imaging acquisition

Cell internalization of fluorescent PNAs was evaluated using BioStation IM (Nikon, Minato, Tokyo, Japan). Until image recording, cells were pre-treated with Hoechst 33342 dye, at final concentration of 0.1 µg/mL, to identify nucleus position into cells. Cells images were taken after 24 hours of incubation using DAPI filter (461 nm) to visualize nuclei and 530 nm filter to visualize FITC conjugate molecules. Two different magnifications were employed x40 and x80.

### RNA Extraction

Cultured cells were trypsinized and collected by centrifugation at 1,200 rpm for 10 minutes at 4 °C, washed with cold DPBS 1X and lysed with Tri-Reagent (Sigma Aldrich, St. Louis, Missouri, USA), according to manufacturer’s instructions. Isolated RNA was washed once with cold 75% ethanol, air-dried and dissolved in nuclease free water before use.

### miRNAs reverse transcription and RT-qPCR

For miRNAs quantification, obtained RNA was reverse transcribed using TaqMan MicroRNA Reverse Transcription Kit (Applied Biosystems, Foster City, CA, USA) and miRNA specific stem-loop primer (*hsa*-miR-96-5p, ID: 000186; *hsa*-miR-155-5p, ID: 002623; *hsa*-miR-210-3p, ID: 000512; *hsa*-miR-221-3p, ID: 000524). Reverse transcription quantitative polymerase-chain reaction (RT-qPCR) was performed according to the manufacturer’s protocols and as indicated elsewhere^50^. All RT reactions, including RT-minus controls and no-template controls were run in duplicate using the CFX96 Touch Real-Time PCR Detection System (BioRad, Hercules, CA, USA). The relative expression was calculated using the comparative cycle threshold method (ΔΔCT) and U6 snRNA (*hsa* U6 snRNA, ID:001973) and *hsa*-let-7c (*hsa*-let-7c, ID:000379) as endogenous controls.

### Analysis of Apoptosis

Analysis of apoptotic profile on U251 cells, transfected with PNAs, was performed using Muse Annexin V & Dead Cell Kit (Millipore, Billerica, MA, USA) according to the instructions supplied by the manufacturer. Briefly, 100 µL of suspension cells were added to 100 µL of Muse Annexin V & Dead Cell reagent, incubated at room temperature, protected from the light for 20 minutes and analysed using Muse Cell Analyzer (Millipore, Billerica, MA, USA).

### Statistical Analysis

Results are expressed as mean ± standard error of the mean (SEM). Comparisons between groups were made by using paired Student’s t test. Statistical significance was defined with p < 0.05 (*, significant) and p < 0.01 (**; highly significant).

## Results

### Analysis of toxicity of 1 on glioma U251 cells

**Figure 2:**
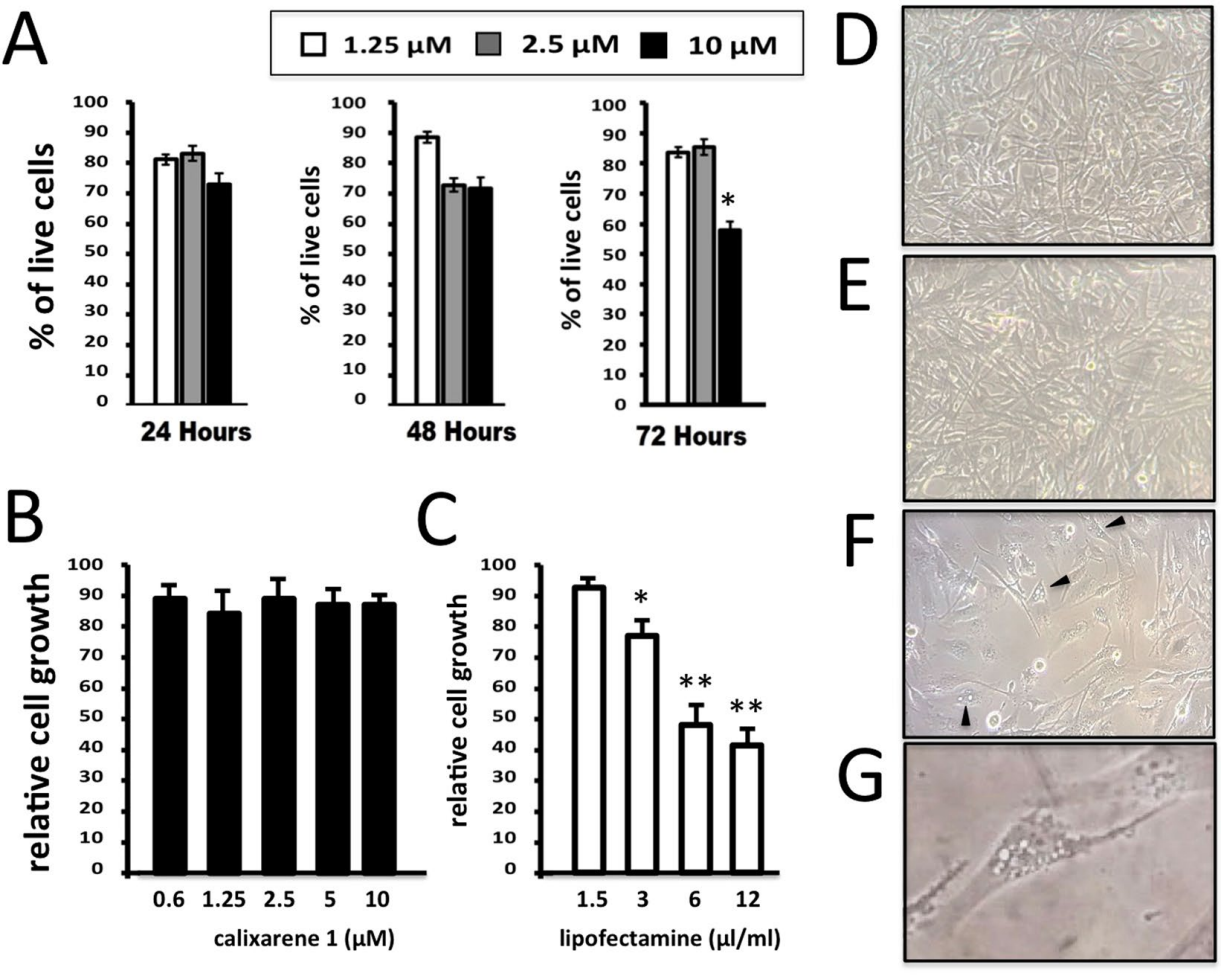
Effects of calixarene **1** on viability, cell proliferation and morphology of U251 cells. (**A**) Viability profile in U251 cells was reported for three incremental concentrations of **1**: 1.25 µM (white bars), 2.5 µM (grey bars) and 10 µM (black bars). Three different time points: 24, 48 and 72 hours after the transfection were considered. (**B**–**G**) Comparison between the effects of calixarene **1** (**B**,**E**) and Lipofectamine RNAiMAX (**C**,**F**,**G**) on U251 cell growth (**B**,**C**) and morphology (**D**–**G**), determined after 72 hours cell culture. D = control untreated U251 cells. Appearance of vacuolization and methuosis-like patterns are underlined by the arrowheads shown in panel F (representative example in the panel G).

One of the major issues associated with the use of commercial available vehicles is the high toxicity of these compounds. For such reason **1** was first evaluated for its effect on cell viability when used to transfect molecules. A viability assay was performed on U251 cell line to detect the rate of live cells, after the transfection with incremental concentrations of transfection agent. Viability profile was determined at three different time points, 24, 48 and 72 hours after transfection, with three different concentrations of **1**, 1.25, 2.5, and 10 μM. Solutions of **1** at these three concentrations were maintained in contact with cells for all the treatment time. As shown in Fig. 2, 24 hours after the transfection no differences in percentage of total live cells were shown when the two lower concentrations (1.25 and 2.5 µM) of **1** were used, while only slight decrease of live cells percentage was detected at 10 µM dosage. The decrease of the proportion of living U251 cells is not unexpected, since it is well established that most of the transfection reagents are to some extent cytotoxic to cells. Interestingly, however, cytotoxic effects of **1** were found only at 10 µM, while for lower concentrations limited effects in cell viability were detected. The effects of **1** on cell growth was determined and shown in Fig. 2B,C.

Comparison was done with the effects of lipofectamine analyzed after 72 hours of cell culture. As clearly evident, lipofectamine causes inhibition of cell proliferation (Fig. 2C), while the anti-proliferative effects of **1** are minor and significant only when the calixarene **1** is used at 10 µM concentration. These findings are in agreement with the cell morphology profile shown in Fig. 2(D–F) and determined by microscopic inspection after 72 hours of cell culture in the presence of 2.5 µM calixarene **1** (Fig. 2E), 3μL/mL lipofectamine (Fig. 2F) or in the absence of both the delivery systems (Fig. 2D). These data support the concept that treatment of the U251 cells with calixarene **1** does not affect cell morphology. On the contrary deep alteration of cell morphology (including shape and shrinking, appearance of vacuolization and methuosis-like phenotypes, toxic granulation) occurs when U251 cells were treated with lipofectamine. The effects of lipofectamine is not unexpected, since this transfection agent has been shown to exhibit cytotoxicity both *in vitro* and *in vivo*^51–55^.

On the basis of the results shown in Fig. 2, all the experiments presented in this study were conducted at 2.5 µM of vector.

### Effects of argininocalixarene 1 on the uptake of calixarene-vehicled PNA by glioma U251 cells

**Figure 3:**
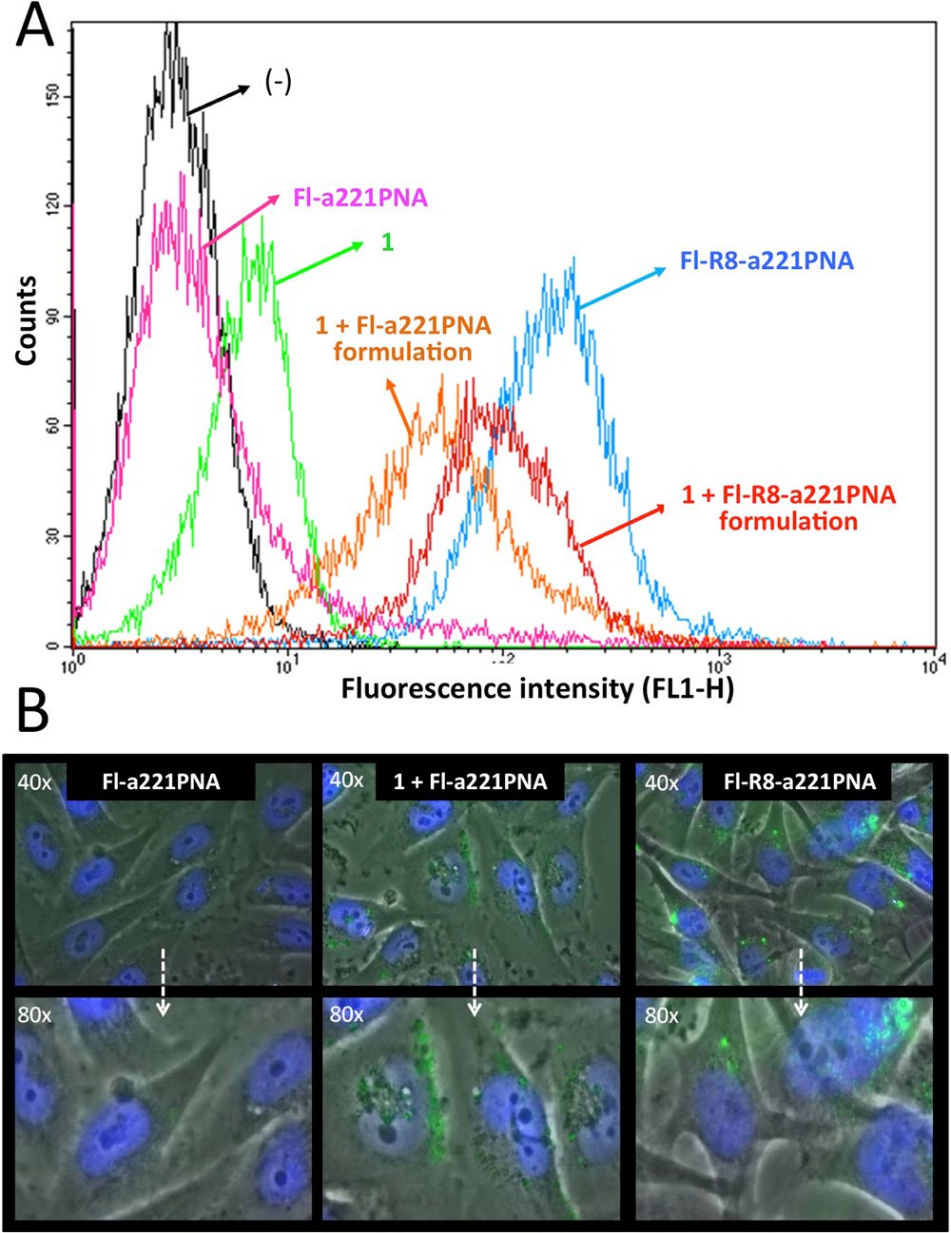
PNA cellular uptake. (**A**) FACS analysis showing the uptake by glioma U251 cells of fluorescein labeled a221PNAs. U251 cells were cultured for 48 hours with the **Fl-a221PNA**, **1/Fl-a221PNA** formulation, **Fl-R8-a221PNA** or **1/Fl-R8-a221PNA** formulation, as indicated. (**B**) Microphotographs showing no accumulation (left), or cytoplasmic accumulation of the delivered PNAs after 24 hours of cell culture.

In order to obtain a proof-of-principle that **1** mediates uptake of PNAs by target cells, a PNA against the microRNA miR-221-3p (**a221PNA**) was employed. The main reason is that **a221PNA** has been extensively studied by some of us and is able to confer to the cells an easily detectable biological end-point, i.e. the induction of apoptosis. In the experiments shown in Fig. 3, a fluorescein labeled version of a221PNA, **Fl-a221PNA**, was used to readily visualize the internalization. First, **1** was loaded with the PNA, in order to obtain a working mixture of **1**/**Fl-a221PNA** at 2.5 and 2 µM, respectively. U251 cells were cultured for 48 hours with the **1**/**Fl-a221PNA** mixture and FACS analysis performed using FACScan. The results obtained are shown in Fig. 3A and indicate that **Fl-a221PNA** delivery mediated by **1** is associated with a complete different distribution of fluorescein-positive U251 cells. In fact, while the naked **Fl-a221PNA** does not exhibit cellular uptake, when it is delivered with **1** almost 100% of the cells were found to be fluorescein-positive. The intensity of the fluorescence signal obtained transfecting **Fl-a221PNA** with **1** approaches that obtained using the free fluorescein labelled **Fl-R8-a221PNA**, including in its structure an octaarginine peptide known to strongly facilitate cellular uptake^50^. The microphotographs depicted in Fig. 3B evidence a cytoplasmic distribution compatible to the data collected by FACS analysis.

A further FACS analysis of fluorescence in cells upon treatment with a mixture of **1** and **Fl-R8-a221PNA** evidenced values between those relative to the **1**/**Fl-a221PNA** mixture and **R8-a221PNA** alone.

**Figure 4:**
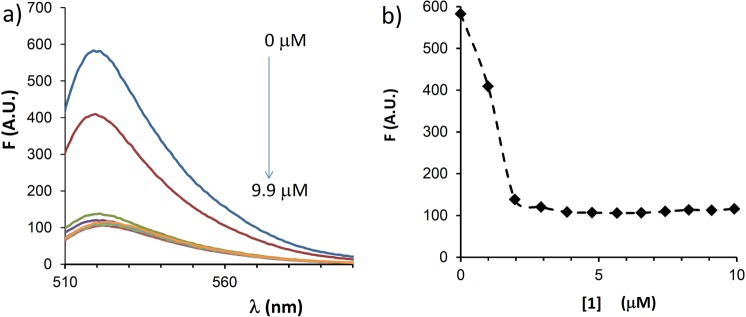
Interaction of PNA with calixarene **1**. Changes in fluorescence intensity of fluorescein labelled **Fl-a221PNA** (2 µM) upon addition of increasing concentration (0, 1.0, 2.0, 2.9, 3.9, 4.8, 5.7, 6.5, 7.4, 8.3, 9.1, 9.9 μM) of calixarene **1** in buffer solution (HEPES 16 mM, NaCl 40 mM, pH 7.4); (**A**) emission spectra of **Fl-a221PNA** (λ_ex_ = 494 nm); (**B**) Fluorescence intensity at maximum vs calixarene concentration.

In order to rule out that the observed delivery was solely due to permeabilization of cells caused by the argininocalixarene, without interaction between the carrier and the PNA, **Fl-a221PNA** was titrated *in vitro* in buffer by adding **1** and measuring the changes in fluorescence emission (Fig. 4). A clear quenching effect was observed, with saturation reached at 2.5 μM concentration of the carrier, after which no further decrease in fluorescence was observed, thus indicating the formation of a complex between carrier and PNA. A slight shift of the fluorescence maximum (from 519 to 523 nm) was also observed, consistent with a more polar microenvironment generated by the cationic calixarene.

### Effects of 1/a221PNA formulation on miR-221

**Figure 5:**
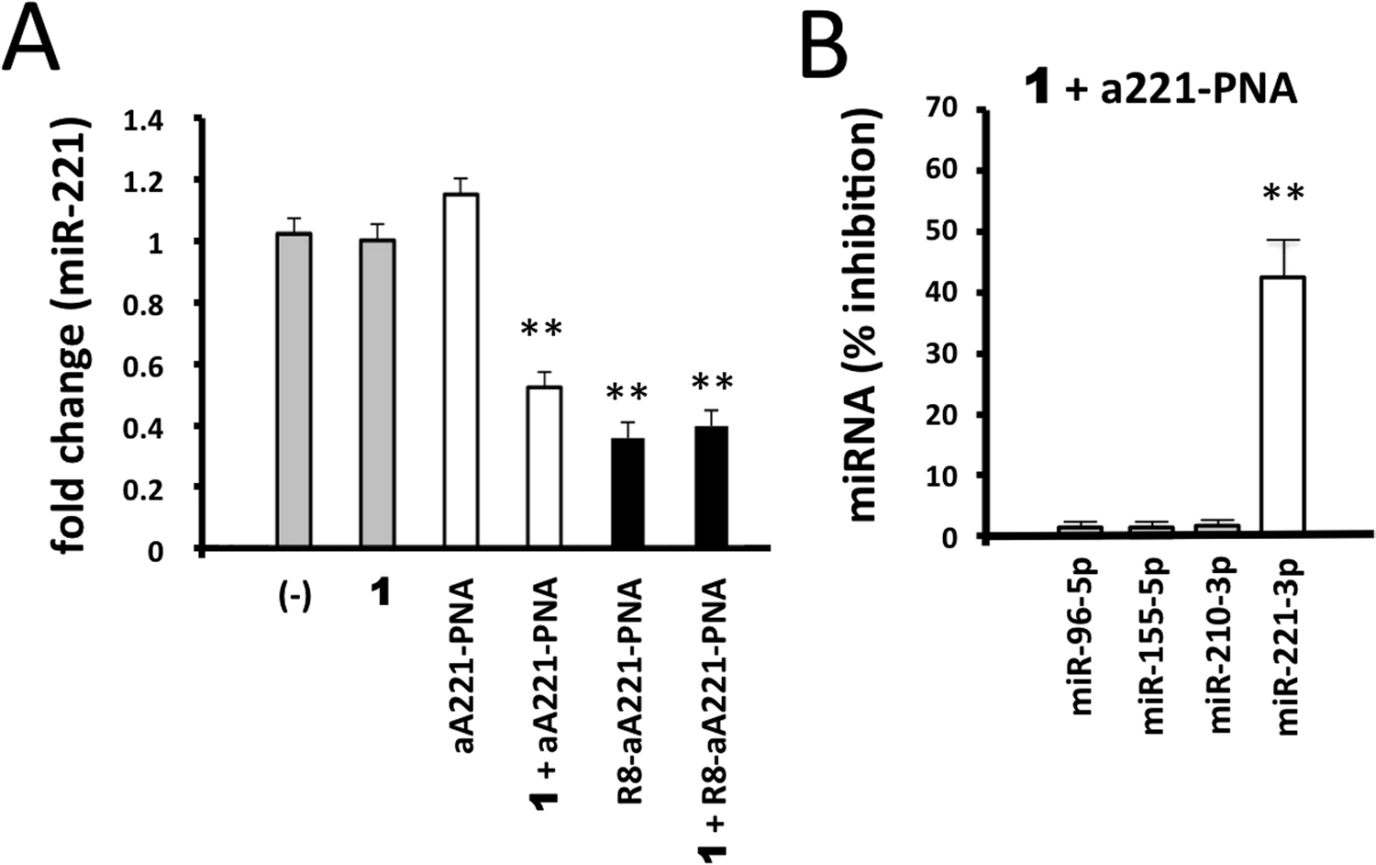
Effects of **1**/a221PNA formulation on miR-221. Glioma U251 cells were treated with **1**, **a221PNA**, **1**/**a221PNA** formulation, **R8-a221PNA**, **1**/**R8-a221PNA** formulation. After 48 hours, RNA was isolated and the hybridization to a miR-221-3p probe determined by RT-qPCR. Calixarene **1** was used at 2.5 μM; PNAs were used at 2 μM.

The data presented in Fig. 3 prompted us to verify whether the biological activity of the **a221PNA** was maintained when delivered with **1**. To this aim we treated glioma U251 cells with **1**, **a221PNA** and **1**/ **a221PNA** formulation. We also considered free **R8-a221PNA** and **1**/**R8-a221PNA** formulation in order to verify possible synergic effects between vector **1** and R8 functionalization. The results of the experiments performed are shown in Fig. 5A and clearly demonstrate that no effects on miR-221-3p hybridization signal was observed in U251 cells treated with **1** alone or with naked **a221PNA** alone. This was expected, since (a) no major changes in gene expression (including miR-221-3p expression) caused by cytotoxicity occurs when 2.5 μM vector **1** concentration is employed, which was demonstrated to be unable to induce alteration of cell viability, morphology and rate of cell growth (see Fig. 2) and (b) **a221PNA** does not have effects on cultured cells, since uptake of this and similar molecules lacking the polyarginine tail is not efficient (see Fig. 3) as also previously reported^50^. On the contrary, significant inhibition of miR-221-3p hybridization was found when U251 glioma cells were treated with **1**/**a221PNA** formulation. Interestingly (a) the efficiency of this inhibition approached that of **R8-a221PNA** and (b) no negative effect of **1** were found in U251 cells transfected with **1**/**R8-a221PNA** formulation.

These data conclusively demonstrate that **a221PNA** delivered by argininocalixarene **1** retains the inhibitory activity on miR-221-3p. The PNA-mediated effects were found to maintain the sequence specificity and selectivity. Despite transcriptomic and proteomic studies will fully clarify possible off-target effects of the proposed delivery system, our results firmly demonstrated that a221PNA delivered by argininocalixarene **1** displayed only minor inhibitory effects on other miRNAs expressed in the glioma U251 cells (such as miR-96-5p, miR-155-5p and miR-210-3p) (Fig. 5B and Supplementary Fig. 1).

### Effects of 1/a221PNA formulation on U251 apoptosis

**Figure 6:**
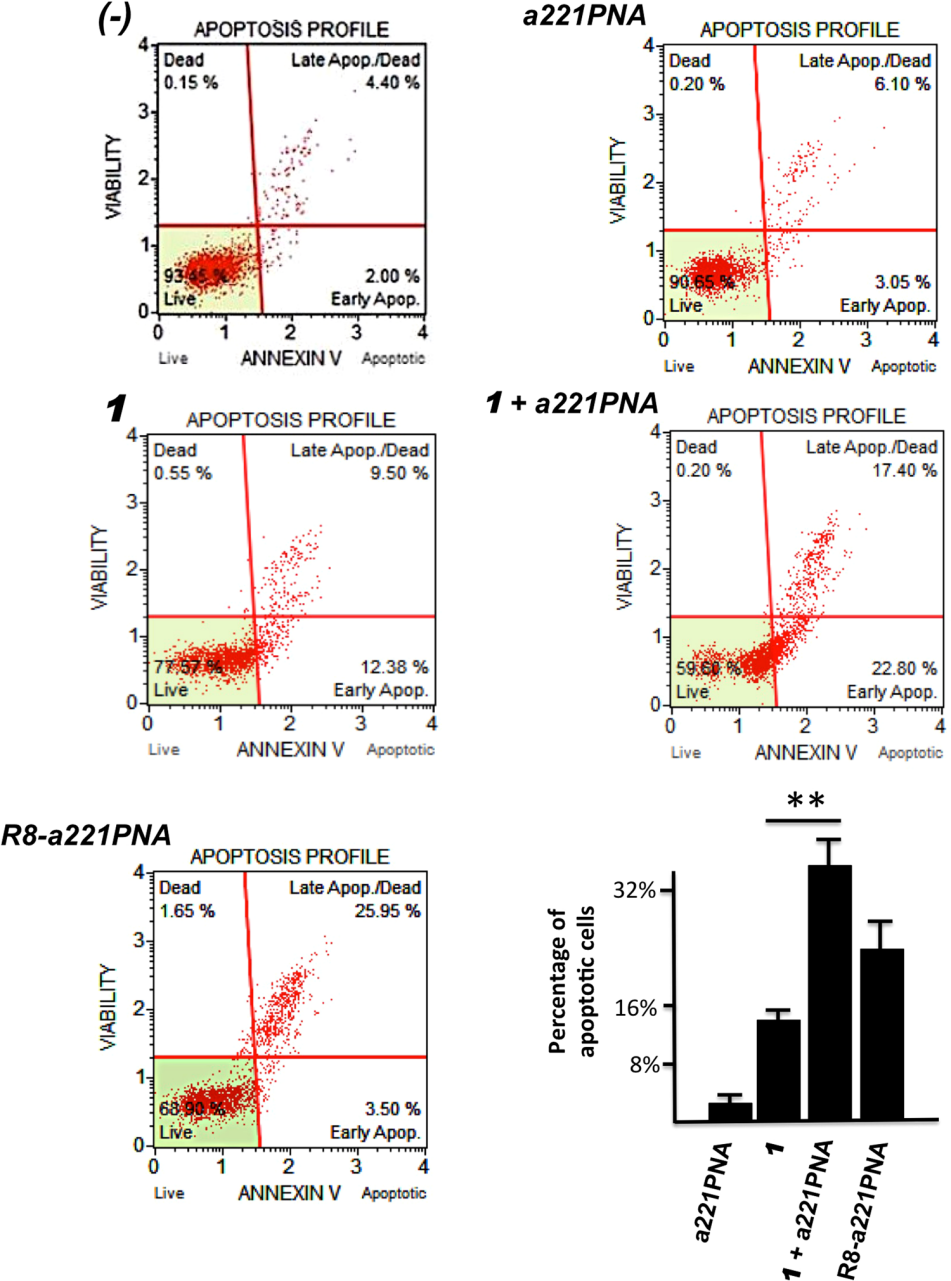
Effects of **1**/a221PNA formulation on U251 apoptosis. U251 cells were cultured with **1**, **a221PNA**, **1**/**a221PNA** formulation, **R8-a221PNA** and **1**/**R8-a221PNA** formulation. After 72 hours the cells were isolated and Annexin-V assay was performed. Percentage of apoptotic cells in bottom graph refers to the sum of early apoptotic and late apoptotic cells.

In order to verify whether **a221PNA** delivered by **1** maintains the known ability of PNA-based molecules targeting miR-221-3p to induce pro-apoptotic effects, U251 cells were cultured for 72 hours as already described in Fig. 5 and the Annexin-V assay was performed. The results are shown in Fig. 6 and clearly demonstrate that **a221PNA** can induce apoptosis only when delivered by **1** or by the covalently linked R8-oligopeptide (as we published elsewhere)^50^. When the values of % apoptosis (including both early apoptotic and late apoptotic cells) of the untreated cells or cells treated only with **1** are subtracted to the values obtained after U251 cell treatment with **R8-a221PNA**, or the **1**/**R8-a221PNA** formulation, respectively, the increase of apoptosis (which, in the representative experiment shown in Fig. 6, was found 23.05% in the case of **R8-a221PNA** and 18.12% in the case of the **1**/**R8-a221PNA)** is fully consistent with the FACS (Fig. 3A) and RT-qPCR (Fig. 5) analyses.

## Discussion

In spite of being among the most interesting and performing oligonucleotide analogues^1–26^, peptide nucleic acids (PNAs) are still of relatively limited use mainly because of the difficulties of internalization into target cells and the lack of a general and simple method for transfection^27–32^. Conjugation with cell penetrating peptides is one of the most used approaches^21,25,33–38^, and several other strategies using nanocarriers^28,29^, polymer particles^40^ and liposomal formulations^39^ have been proposed. To boost research and application of these compounds, the availability of a carrier molecule that can be simply mixed in the incubation medium with unmodified PNAs would be of great relevance.

The data here reported indeed indicate that argininocalix[4]arene **1**^46^ can represent this type of delivery system. It is able to efficiently transport neutral PNA molecules to target cells while preserving their biological activity, after a very simple formulation procedure. The following conclusions can be drawn from the obtained results: (a) since the toxicity of **1** is low (see Fig. 2), it can be proposed for long-term treatment of target cells, being this feature a pre-requisite for the development of therapeutic protocols; (b) the delivery of PNAs is efficient, being comparable to that of PNAs functionalized with cell penetrating peptides (see Fig. 3); (c) the biological activity of PNA delivered using **1** is maintained (see Figs 5 and 6).

Of great relevance in our opinion is that the efficiency of the increase of the proportion of apoptotic cells when U251 cells are treated with **1**/**a221PNA** formulation is comparable to that obtained using **R8-a221PNA**.

The localization inside the cells of the fluorescently labelled **Fl-a221PNA** delivered by **1** is more diffused compared to that of the peptide-conjugated **Fl-R8-a221PNA**, suggesting that endosomal escape is more efficient for the calixarene-delivered PNA and thus enhancing the effectiveness of the PNAs in cellular systems.

No synergy on the transfection process were observed by mixing **1** with R8 containing a221PNA. The FACScan test evidenced a fluorescence similar to that of **Fl-R8-a221PNA** alone (Fig. 2) and the effect on miR-221-3p resulted comparable with that of **R8-a221PNA** transfected alone. This could be due to repulsive interactions between the arginines present both in **1** and **R8-a221PNA** that do not allow the binding between the two molecules and at the same time disfavor the delivery of **R8-a221PNA** alone.

The significant effect of the calixarene on the uptake of a221PNAs is not merely attributable to a cell permeabilization effect, but it can be related to a strong binding to the PNA, as revealed by the titration experiments depicted in Fig. 4. This supports the initial hypothesis that argininocalixarene **1** can establish other types of interactions with oligonucleotides and oligonucleotide analogues different from the electrostatic ones with phosphate groups, as actually verified for other cationic calixarene derivatives with nucleic acids^47,48^. Moreover, it is known that in protein-DNA complexes, positively charged amino acid side chains not only interact with negative charges, but give rise to other specific interactions such as hydrogen bonding, π-π and cation-π interactions with nucleobases^56,57^. Analogously, in view of the neutral nature of the PNA backbone hydrogen bonding and cation-π interactions could realistically occur to a different extent between guanidinium and/or ammonium groups of vector **1** and different nucleobases possibly being more intense with electron-rich purines, in particular guanine. It is likely that multiple different interactions concur to the formation of the PNA:calixarene adduct; thus the exact stoichiometry and strength of binding of other PNAs cannot be extrapolated at this stage. These interactions would constitute the first step of the mechanism of transport of PNA by calixarene **1**. On the basis of the mode of binding to and condensation of DNA proposed for guanidinocalixarenes^43^ and of the experiments in this study, it is in fact reasonable to think that subsequently, besides these specific interactions, a series of intermolecular hydrophobic interactions among the alkyl chains of different calixarenes bound to PNA molecules occur during the transport into cytosol across the cellular membrane. In any case, though studies are still necessary to shed light on this aspect, it is evident that the PNA:calixarene interactions take place rapidly and efficiently, thus greatly simplifying the transfection formulation protocol.

These findings indicate argininocalix[4]arene **1** as unique molecule for efficient delivery of PNAs to target cells. Its peculiarities are the ease of preparation, the relative structural simplicity associated to high delivery efficiency and negligible toxicity. Although the efficiency of uptake by cells for PNA delivered by **1** is not higher but comparable with that of PNA functionalized with R8 peptide, its use results much more convenient because avoids any modification on the PNA molecules intended to be delivered and the transfection formulation is simply attained by mixing vector and PNA. Calixarene **1** therefore could represent a universal vector for all unmodified PNAs that can simplify and boost their study and use as antisense, and possibly anti-gene, derivatives.

## Supplementary information

Supplementary Figure 1
