# Supplementary material for: Efficient cell penetration and delivery of peptide nucleic acids by an argininocalix[4]arene
Source: Sci Rep. 2019 Feb 28;9:3036. doi: 10.1038/s41598-019-39211-4 (PMC6395679; doi:10.1038/s41598-019-39211-4)
Supplement: Supplementary file 1 — Supplementary Figure 1 [file 41598_2019_39211_MOESM1_ESM.pdf]

Efficient cell penetration and delivery of peptide nucleic acids by an argininocalix[4]arene

Jessica Gasparello‡, Alex Manicardi†, Alessandro Casnati†, Roberto Corradini†, Roberto Gambari‡, Alessia Finotti‡*, Francesco Sansone†*

‡Department of Life Sciences and Biotechnology, Section of Biochemistry and Molecular Biology, University of Ferrara, Italy;

†Department of Chemistry, Life Sciences and Environmental Sustainability, University of Parma, Italy.

Supporting Information

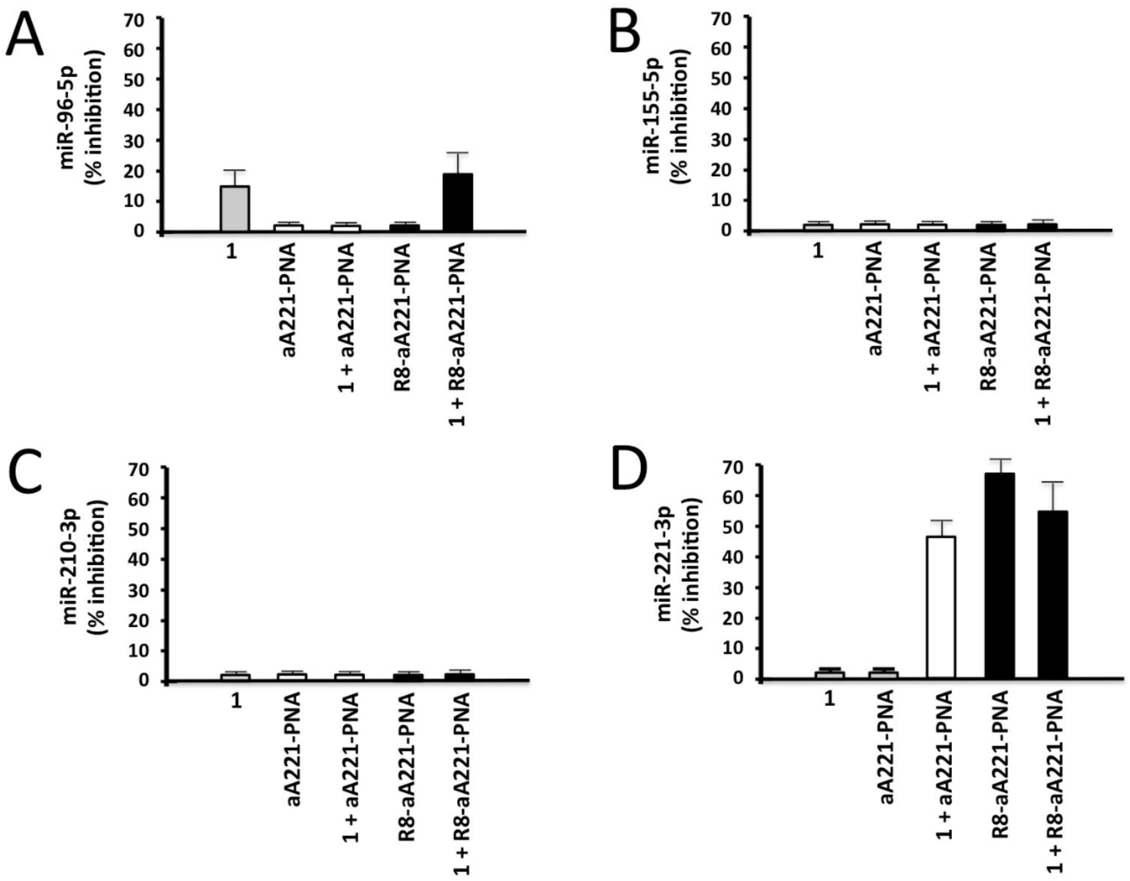

Supplementary Figure 1. Effects of 1/a221PNA formulation on miR-96-5p, miR-155-5p, miR-210-3p and miR-221-3p. Glioma U251 cells were treated with **1**, **a221PNA**, **1**/**a221PNA** formulation, **R8-a221PNA**, **1**/**R8-a221PNA** formulation. After 48 hours, RNA was isolated and the hybridization to miR-96-5p, miR-155-5p, miR-210-3p and miR-221-3p probes determined by RT-qPCR. Calixarene **1** was used at 2.5 μM; PNA molecules were used at 2 μM.
